# CREATING AN AGE-FRIENDLY UNIVERSITY: THE CASE OF ST. CLOUD STATE UNIVERSITY (SCSU)

**DOI:** 10.1093/geroni/igac059.1723

**Published:** 2022-12-20

**Authors:** Phyllis Greenberg, Rona Karasik, Jessica VanderWerf, Claudia Burgos Zuniga, Maria Kroeber

**Affiliations:** St. Cloud State University, St Cloud, Minnesota, United States; St. Cloud State University, St. Cloud, Minnesota, United States; St. Cloud State University, St. Cloud, Minnesota, United States; St. Cloud State University, St. Cloud, Minnesota, United States; St. Cloud State University, St. Cloud, Minnesota, United States

## Abstract

As universities seek ways to address decreasing enrollments and enhance Diversity, Equity, and Inclusion (DEI) initiatives, the concept of Age-Friendly Universities (AFU) has gained momentum. The AFU Global Movement is a way to situate a university to explore means to increase student enrollments, and expand DEI Initiatives to include age. This poster details the steps taken by St. Cloud State University, a comprehensive Minnesota State university, to become designated as an AFU, as well as the unique challenges encountered. Actions taken both before and after being designated as an Age Friendly University (December, 2021) are discussed. Pre-designation steps included evaluating the campus’ current age friendliness, gathering university and community support at all levels, participating as a “champion” in the UMass-Boston & LaSalle University’s Age-Friendly University Inventory and Campus Climate survey, and piggy-backing on Minnesota’s Age-Friendly State and the local community’s Age-Friendly initiatives. The SCSU Gerontology program was awarded a grant from the MinnState Innovation Fund to explore ways of “Creating an Age-Friendly SCSU.” The grantors saw this as a potential template for other universities in the system who were seeking to expand their student base. Post benefits, challenges, strategies and next steps working towards meeting the 10 Principles of an Age-Friendly University are also discussed.

